# *Deinococcus radiodurans* UWO298 Dependence on Background Radiation for Optimal Growth

**DOI:** 10.3389/fgene.2021.644292

**Published:** 2021-05-06

**Authors:** Hugo Castillo, Xiaoping Li, Geoffrey B. Smith

**Affiliations:** ^1^Human Factors and Behavioral Neurobiology Department, Embry–Riddle Aeronautical University, Daytona Beach, FL, United States; ^2^Virginia Tech Hampton Roads Agriculture Research and Extension Center, Virginia Tech, Blacksburg, VA, United States; ^3^Department of Biology, New Mexico State University, Las Cruces, NM, United States

**Keywords:** background radiation, transcriptome analysis, stress response, homeostatic control, *de novo* transcriptome analysis

## Abstract

Ionizing radiation is a major environmental variable for cells on Earth, and so organisms have adapted to either prevent or to repair damages caused by it, primarily from the appearance and accumulation of reactive oxygen species (ROS). In this study, we measured the differential gene expression in *Deinococcus radiodurans UWO298* cultures deprived of background ionizing radiation (IR) while growing 605 m underground at the Waste Isolation Pilot Plant (WIPP), reducing the dose rate from 72.1 to 0.9 nGy h^–1^ from control to treatment, respectively. This reduction in IR dose rate delayed the entry into the exponential phase of the IR-shielded cultures, resulting in a lower biomass accumulation for the duration of the experiment. The RNASeq-based transcriptome analysis showed the differential expression of 0.2 and 2.7% of the *D. radiodurans* genome after 24 and 34 h of growth in liquid culture, respectively. Gene expression regulation after 34 h was characterized by the downregulation of genes involved in folding newly synthesized and denatured/misfolded proteins, in the assimilation of nitrogen for amino acid synthesis and in the control of copper transport and homeostasis to prevent oxidative stress. We also observed the upregulation of genes coding for proteins with transport and cell wall assembly roles. These results show that *D. radiodurans* is sensitive to the absence of background levels of ionizing radiation and suggest that its transcriptional response is insufficient to maintain optimal growth.

## Introduction

The role of background ionizing radiation (IR) as an environmental cue in cells has been previously documented in various models that include multicellular (Planel et al., 1987; Kawanishi et al., 2012; Van Voorhies et al., 2020) and unicellular (Satta et al., 1995) eukaryotes, mammalian cell cultures (Satta et al., 2002; Fratini et al., 2015), and bacteria (Planel et al., 1987; Smith et al., 2011; Castillo et al., 2018). Regardless of their taxonomic and physiological differences, most of these models showed, to varying degrees, deleterious effects attributable to their growth under IR dose rates below natural background levels. Although the molecular mechanisms for this unexpected effect are not yet fully understood, there is a growing body of evidence that all cells rely on a minimum physiological concentration of ionizing radiation-derived reactive oxygen species (ROS) to control basic processes such as gene regulation, DNA repair, and growth (Murphy et al., 2011; Ray et al., 2012; Imlay, 2013).

*Deinococcus radiodurans* belongs in a phylum widely distributed in nature, occupying niches from deep ocean subsurface and hot springs to arid desert soils (Daly, 2009), and it is understood that its ability to survive acute exposure to ionizing radiation (∼17 kGy) is likely due to its niche of high temperature and low water availability (Mattimore and Battista, 1996; Rainey et al., 2005). This adaptation is the result of cellular and molecular mechanisms that protect its proteins and DNA from extreme degradation and to the modification of its proteins to prevent degradation and to retain catalytic activity for metabolic and DNA repair processes (Liu et al., 2003). For example, compared with radiation-sensitive *Escherichia coli*, *D. radiodurans*’ RecA is more efficient repairing double-stranded DNA breaks (Pobegalov et al., 2015), has at least four layers in its thick cell wall (Brooks and Murray, 1981), and its DNA is organized in a toroidal conformation (Englander et al., 2004). Lim et al. (2019) have found a surprising diversity of radiation-resistant genes that are spread across *Deinococcus* species, whereas regulatory genes that control radiation/desiccation regulons were conserved.

Another radiation resistance strategy in *Deinococcus radiodurans* is its accumulation of anti-oxidant metals like Mn(II), which protects its proteins from radiolytic reactive oxygen species (Daly et al., 2004). As discussed by Daly (2009), proteins, and not DNA, may be the more biologically critical target of ionizing radiation since it is through enzymes that single- and double-stranded breaks are repaired since radiation-resistant organisms undergo as much DNA damage as radiation-sensitive organisms (Daly et al., 2004; Daly, 2009). Similarly, the DNA repair enzymes of *D. radiodurans* are not significantly different than radiation sensitive prokaryotes (Daly, 2009).

The first *D. radiodurans* species was discovered in the 1950s, with the R1 type strain isolated from an irradiated canned meat container and the Sark strain identified as a lab contaminant (Anderson et al., 1956). Later on, Brooks et al. (1980) analyzed DNA hybridization melting curves of various strains of *D. radiodurans*, finding only a 33% homology between R1 and Sark (respectively, called *UWO 288* and *UWO 298*), and yet concluded the two to be members of the same species. As part of an effort to solve this discrepancy, Rainey et al. (1997) sequenced the 16S rDNA of the two strains and found them to have a 96.9% sequence similarity, noting that the low DNA homology suggested that these two strains were members of distinct genospecies. These two strains are the only *D. radiodurans* strains maintained by ATCC, *UWO 288* (R1) as ATCC 13939 and the Sark strain, *UWO 298*, as ATCC 35073. The transcriptome data presented here exemplifies the disparate chromosomal DNA homology documented by Brooks et al. (1980) as the initial read alignment of our libraries from *UWO 298* cultures against the reference strain (R1) exhibited a low RNA read alignment supporting the findings by Brooks and Rainey.

We have previously reported a growth deceleration effect on *D. radiodurans* with the concomitant lower biomass accumulation in a 48-h culture, in multiple experiments of the Low Background Radiation Experiment (LBRE) at the Waste Isolation Pilot Plant (WIPP), described elsewhere (Castillo and Smith, 2017). In summary, *D. radiodurans* cultures were grown at IR dose rates of 0.91 and 72.05 nGy h^–1^ as treatment and control, respectively. In order to explore the underlying causes of the difference in growth dynamics, we documented the differential expression of four stress-related genes, which suggested the inability of this organism of a timely transcriptional response to retain optimal growth (Castillo and Smith, 2017). However, our criteria for the selection of the target genes were the direct comparison to the response previously observed in *Shewanella oneidensis* (Castillo et al., 2015) and, therefore, may not have been the better descriptors for this taxonomically unrelated species. Here we present the results from the *de novo* transcriptome analysis of the Sark strain of *D. radioduran*s grown under the IR-shielded conditions at WIPP (Castillo and Smith, 2017).

## Materials and Methods

### *Deinococcus radiodurans* Growth, Growth Measurement, and Sampling

*Deinococcus radiodurans* (ATCC 35073) cultures were grown, in triplicate and at background conditions, in 2 ml of TGY (tryptone–glucose–yeast extract) media under constant agitation (150 rpm) at 30°C (from here on referred to as standard conditions). The inoculum for the experiment (20 μl) was transferred into 2 ml of TGY and incubated overnight under the previously described conditions (Castillo et al., 2018). This overnight culture was then brought underground to the WIPP laboratory where it was further diluted to approximately 3 × 10^7^ cells per ml. From this cell suspension, 1.5 ml was transferred into the top row of the 24-well plate and grown at a dose rate of either 72.1 nGyhr^–1^ (control) or 0.91 nGyh^–1^ (treatment), on an orbital shaker at 200 rpm during 24 h at 30°C. After this initial growth period, a sample pooled from the first four wells was then diluted to 1:50, and 1.5-ml aliquots were transferred to the second row of the plate to re-initiate their growth under the same conditions. Growth was estimated measuring optical density at 630 nm using a microplate reader (ELX800, Biotek, Winooski, VT, United States) at 10, 24, 29, 34, and 48 h. After measurement, 300 μl from two wells was pooled and mixed with 600 μl of RNAprotect (QIAGEN, Valencia, CA, United States) and frozen at −20°C until transported to the surface lab for further processing.

### RNA Library Preparation

In preparation for RNA library construction, total RNA was extracted from samples collected at 24 and 34 h using the RNAeasy QIAGEN kit (QIAGEN, Valencia, CA, United States) according to the protocol provided by the manufacturer, including a DNAse I incubation to eliminate traces of genomic DNA. Prior to library construction, total RNA concentration and integrity were estimated using the RNA Qubit assay (Invitrogen, Burlington, ON, Canada) and the Bioanalyzer RNA pico assay (Agilent Technologies, Santa Clara, CA, United States), respectively. Ribosomal RNA was removed with the RiboZero kit for bacteria, and the eluted mRNA was purified with the RNAClean XP kit (Beckam Coulter, Beverly, MA, United States). The RNA libraries were constructed with the ScriptSeq Complete kit for bacteria (Epicentre, Madison, WI, United States) following the manufacturer’s instructions. After PCR amplification of the cDNA, the samples were purified using the Agencourt AMPure XP system (Beckam Coulter, Beverly, MA, United States). Quantification of the libraries was performed with the AMPure XP system (Beckam Coulter, Beverly, MA, United States), and the fragment distribution was estimated with the Bioanalyzer High Sensitivity DNA assay (Agilent Technologies, Santa Clara, CA, United States). Three libraries from each treatment and timepoint were sequenced using the HiSeq 200 Illumina platform at the National Center for Genome Research in Santa Fe, NM.

### Raw Read Quality Processing and Trinity *de novo* Assembly

Raw 50-bp long single-end reads were first subjected to Trimmomatic v0.36 (Bolger et al., 2014) (ILLUMINACLIP:2:30:10 LEADING:3 TRAILING:3 SLIDINGWINDOW:4:15 MINLEN:36) to remove adapter sequences and unknown (N’s) and low-quality bases. The processed reads were screened using FastQC^1^ to evaluate the overall qualities. *De novo* assembly was performed using Trinity v2.4.0 (Grabherr et al., 2011) on the server service provided by the New Mexico State University Computer Science department using all the processed reads from 12 libraries (Supplementary Table 1). The assembly was further screened with the NCBI Contamination Screen^2^ for quality control. Transcripts less than 200 bp and six sequences considered as contamination were excluded. In addition, 27 sequences were trimmed for undetected adapter sequences by customized R scripts (Supplementary Table 2). To reduce the redundancy, CD-HIT v4.6^3^ was applied to extract the transcripts with longest ORF, from here on referred to as unigenes, with parameters set at -c: 0.9 -n 8 -M 16000 -T 2 -r 1. All the downstream analyses were based on the unigenes.

### Assembly Quality Assessment, Gene Functional Annotation, Differential Gene Expression Analysis, and GO Term Enrichment

The statistics of the assessment, including transcript length coverage, RNA-Seq read representation, and Contig-Nx status on the assembly were generated by the programs and command lines provided by Trinity (Grabherr et al., 2011). The functional annotation of unigenes was accomplished using the Swiss-prot-based Trinotate v3.0.2^4^ suite that integrates the eggNOG/GO/KEGG databases and searches unigenes by well-referenced methods such as BLAST, HMMER/Pfam, SignalP, and tmHMM to retrieve data on homology, protein domain identification, protein signal peptide, and transmembrane domain prediction. Differentially expressed genes were ran against this file to retrieve annotations with gene id.

In order to assess differential expression of the genes at 24 and 34 h, estimation of transcript abundance was first carried out with alignment-based RSEM method using the toolkit provided with Trinity. The Trinity assembly served as reference sequences, and the processed reads from all libraries were separately aligned back to the assembly using Bowtie2 (Langmead and Salzberg, 2012). The quantification of the read counts was normalized to the count number to FPKM and TPM, and it generated two files containing the count number, FPKM, and TPM of each transcript for genes and isoforms. Trinity gene count matrices combining samples from 24- and 34-h libraries were built, respectively. The count matrices were then uploaded into DESeq2 (Love et al., 2014) in R for the statistical analysis of differential expression. The threshold for the determination of significantly expressed genes were set at log_2_ fold change >1 or <−1 and FDR ≦ 0.1.

All GO term assignments for each gene feature were extracted from the Trinity annotation report using a provided perl script *extract_GO_assignments_from_Trinotate_xls.pl* in Trinotate. The length file for each unigene was obtained by customized python script. Files containing expressed genes (fold change > 1 or <−1) for 24 and 34-h samples were generated and used as inputs to perform GO term enrichment analysis using the Bioconductor package GOseq (Young, 2010).

### qPCR Validation

In order to validate our transcriptome results using qPCR, total RNA was reverse transcribed using the SuperScript^TM^ IV First Strand Synthesis System (Invitrogen, Burlington, ON, Canada) following the manufacturer’s instructions. Quantitative PCR was performed, in triplicate, using the Applied Biosystems^TM^ PowerUp^TM^ SYBR^TM^ Green Master Mix (Waltham, MA, United States). Ten-microliter reactions were set up with 5 μl of the PowerUP SYBR green 2X master mix, 1 μl of each forward and reverse primers, specifically designed for this study (Supplementary Table ST1), and 5 ng of template cDNA, using the standard cycling mode (primer Tm < 60°C) recommended in the Mastermix user’s guide. The relative expression of each target gene was calculated with the 2^–ΔΔCt^ equation (Livak and Schmittgen, 2001) using two regions of the 16S rRNA gene (primers 16A and 16B) as reference genes for normalization.

## Results

**FIGURE 1:**
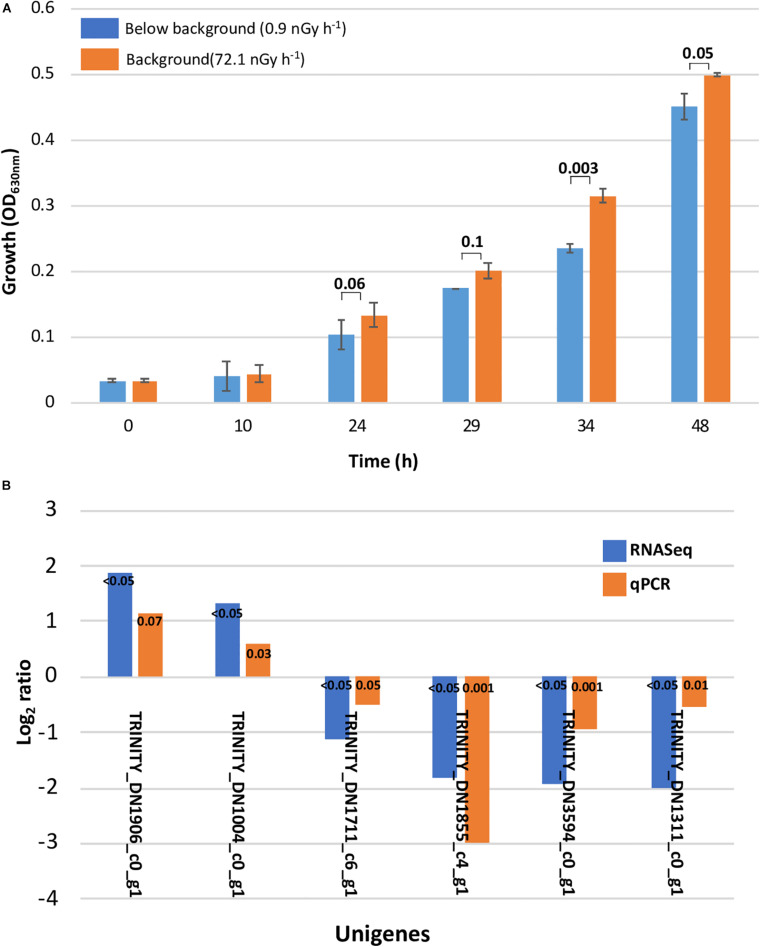
Slower growth and qPCR validation. **(A)** Slower growth in *Deinococcus radiodurans* cultures grown in the absence of background levels of ionizing radiation. Bars show standard error bars and a *p*-value significance number. **(B)** Transcriptome analysis validation with differential gene expression calculated using qPCR. The unigenes used as target and reference and their function are described in Supplementary Table 1 (ST1).

The DNA homology between the UWO298 strain and the R1 strain reference genome was indicated at approximately 33%, using Rockhopper for reads alignment (McClure et al., 2013). Due to the high degree of dissimilarity, the published genome of the R1 type strain could not be used as a template, and so a *de novo* method was implemented to assemble all the reads from the 12 Sark libraries using Trinity (Grabherr et al., 2011). The present study shows a growth deceleration response in *D. radiodurans* cultures directly related to an artificial dose of ionizing radiation approximately 80 times lower than background levels (Figure 1A). In order to explore the underlying molecular mechanisms leading to this effect, we measured the differential transcriptional regulation of *D. radiodurans* in liquid cultures grown for 24 and 34 h under the conditions previously described and reported (Castillo and Smith, 2017). For this purpose, 12 libraries of *D. radiodurans UWO 298* sequenced by Illumina HISEQ2000 platform, generated a total of 50-bp singled-end 204.7 million reads. The reads from both timepoint libraries were deposited in the NCBI SRA repository under the accession number PRJNA389981. Since the read alignment rate using the ATCC 13939 genome as reference was estimated at approximately 33%, using Rockhopper for read alignment (McClure et al., 2013), a *de novo* method was implemented to assemble all the reads from 12 libraries using Trinity (Grabherr et al., 2011). A total number of 204.7 million raw reads were generated and subjected to the processing of Trimmomatic and quality control. This yielded approximately 191.9 million trimmed reads (Supplementary Table ST2) for assembly.

**FIGURE 2:**
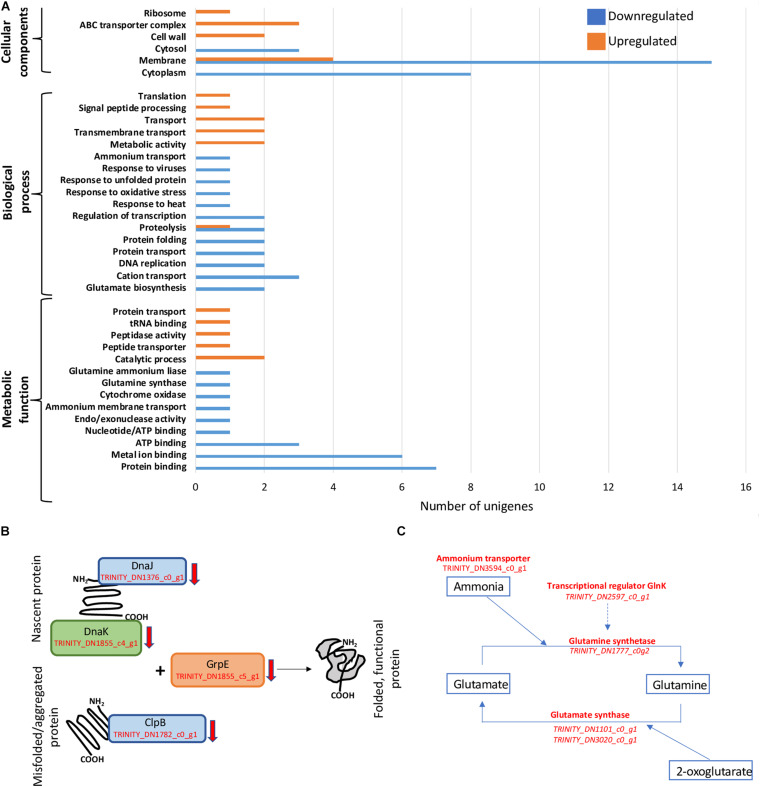
General patterns of regulation and decrease in transcription of genes involved in protein folding and nitrogen acquisition. **(A)** Gene Ontology (GO) term enrichment analysis of *D. radiodurans*’ 34-h cultures. **(B)** Downregulation of genes involved in protein folding and re-folding. **(C)** Downregulation of N-assimilation-related genes.

The original Trinity *de novo* assembled 3,076 transcripts with a length of 2,872,148 nucleotides and yielded 4,796 contigs with N50 of 1,486 bp. Additional quality control excluded six contaminated sequences and trimmed 27 sequences containing adapters according to the NCBI contamination report. Transcript length equal or larger than 200 bp were retained. We implemented CD-HIT-EST to obtain unigenes by clustering transcripts with 90% identity. This resulted in a total number of 3,032 unigenes with N50 of 1,486 bp, approximating the 3,167 protein-coding genes reported for the reference strain genome (White et al., 1999). Mapping trimmed reads to the assembly reports 65% of unique alignment rate for a 24-h library with overall alignment rate of 68%. For a 34-h library, 68% of unique alignment rate and an overall alignment rate of 72% was reported (Supplementary Table ST3). According to our *de novo* transcriptome analysis, the subtraction of background levels of radiation from our treatment cultures resulted in 7 and 88 significantly regulated unigenes after 24 and 34 h, respectively, which corresponds to 0.2 and 2.7% of the genome. At 24 h, all of the unigenes were downregulated, whereas at 34 h, 24 were upregulated and 64 were downregulated (Table 1). The validity of our transcriptome analysis was confirmed using a subset of genes amplified with RT-qPCR and compared with the expression levels obtained with RNASeq (Figure 2).

**TABLE 1:** 

**Unigene**	**BLASTx**	**Log2**	**FDR**	**Molecular function**	**Biological process**	**Cellular component**
**24 h**						
TRINITY_DN165_c0_g1	Transposase for insertion sequence-like element IS431mec	−1.44	4.20E−07			
TRINITY_DN1209_c0_g1,	Putative ring-cleaving dioxygenase MhqO	−1.37	2.46E−03			
TRINITY_DN2566_c0_g1	Heme-binding protein A	−1.31	2.46E−03	Metal ion binding	Proteolysis	
TRINITY_DN939_c0_g1	*N*-acetylmuramic acid 6-phosphate etherase	−1.20	3.41E−03	Carbon–oxygen lyase activity	Peptidoglycan turnover	
TRINITY_DN1650_c0_g1	Probable ABC transporter-binding protein DR_1571	−1.19	2.46E−03	Peptide transmembrane transporter	Transmembrane transport	ABC transporter
TRINITY_DN1780_c0_g3	NAD(P)H dehydrogenase (quinone)	−1.15	2.51E−03	Oxidoreductase activity	Oxidation–reduction process	
TRINITY_DN4285_c0_g1	Anhydro-*N*-acetylmuramic acid kinase	−1.05	3.01E−02	Carbon–oxygen lyase activity	Peptidoglycan turnover	
**34 h**						
TRINITY_DN1209_c0_g1	Putative ring-cleaving dioxygenase MhqO	−3.51	1.59E−38			
TRINITY_DN1787_c0_g1	18 kDa heat shock protein	−2.22	7.40E−13		Protein transport	membrane
TRINITY_DN1777_c0_g2	Type-3 glutamine synthetase	−2.12	1.95E−05	Glutamine–ammonia ligase activity	Nitrogen metabolic process	
TRINITY_DN1311_c0_g1	Copper-transporting ATPase 1	−2.01	1.54E−09	Metal ion binding	Cation transport	Membrane
TRINITY_DN1782_c0_g1	Chaperone protein ClpB	−2.01	1.63E−07	Protein binding, ATPase activity	Response to unfolded protein	Cytoplasm
TRINITY_DN1822_c1_g1	Hemin ABC transporter substrate-binding protein	−1.98	1.74E−06			
TRINITY_DN4285_c0_g1	Anhydro-N-acetylmuramic acid kinase	−1.98	1.24E−06	ATP binding, kinase activity	Carbohydrate metabolic process	
TRINITY_DN2957_c0_g1	Nitrogen regulatory protein P-II	−1.96	1.70E−05	Protein binding, enzyme regulator activity	Regulation of transcription	Cytosol
TRINITY_DN939_c0_g1	N-acetylmuramic acid 6-phosphate etherase	−1.95	1.24E−06	Protein binding	Peptidoglycan turnover	
TRINITY_DN3594_c0_g1	Ammonia channel	−1.94	2.71E−03	Ammonium transmembrane transport activity	Ammonium transport	Membrane
TRINITY_DN2566_c0_g1	Heme-binding protein A	−1.94	4.32E−11	Metal ion binding activity	Proteolysis	
TRINITY_DN1552_c0_g1	Uncharacterized protein YkwD	−1.88	6.59E−10			
TRINITY_DN1855_c4_g1	Chaperone protein DnaK	−1.80	3.23E−07	Protein binding, ATPase activity	Protein folding, DNA replication	Cytoplasm
TRINITY_DN1511_c0_g1	Oligopeptide transport system permease protein OppC	−1.75	8.02E−10		Protein and peptide transport	Membrane
TRINITY_DN1650_c0_g1	Probable ABC transporter-binding protein DR_1571	−1.70	4.32E−11	Metal ion binding, electron transfer activity	Electron transport chain	Membrane
TRINITY_DN1535_c0_g1	AI-2E family transporter	−1.70	1.42E−04	ATPase activity, transmembrane movement	Transmembrane transport	Membrane
TRINITY_DN1005_c0_g1	CRISPR-associated helicase Cas3	−1.69	1.54E−09	Endo/exonuclease activity	Response to virus	Cytoplasm
TRINITY_DN1794_c1_g4	Hypothetical protein BsLM_0616	−1.68	5.73E−04			
TRINITY_DN1822_c2_g1	Hemin import ATP-binding protein HmuV	−1.64	3.38E−04	ATP binding, ATPase activity	Transport	Membrane
TRINITY_DN1780_c0_g3	NAD(P)H dehydrogenase (quinone)	−1.61	6.59E−10	Nucleotide and NAD(P)H dehydrogenase activity	Response to oxidative stress	
TRINITY_DN895_c0_g1	Glycosyl hydrolase	−1.60	1.57E−06			
TRINITY_DN1807_c0_g1	Hypothetical protein A15U_04539, partial	−1.56	2.36E−03			
TRINITY_DN1653_c0_g1	Dehydration responsive domain protein, partial	−1.54	4.27E−03			
TRINITY_DN1519_c0_g1	IS1 transposase InsAB, partial	−1.54	2.61E−02			
TRINITY_DN1170_c0_g1	Hypothetical protein HMPREF9610_00001, partial	−1.53	6.78E−02			
TRINITY_DN610_c0_g1	Copper-transporting ATPase 2	−1.52	4.16E−04	Metal ion binding	Cation transport	Membrane
TRINITY_DN1805_c1_g2	RNAase	−1.52	1.59E−03			
TRINITY_DN1101_c0_g1	Ferredoxin-dependent glutamate synthase 1	−1.52	1.24E−06	Glutamate synthase activity	Glutamate biosynthetic process	Cytoplasm, membrane
TRINITY_DN1554_c0_g1	Probable plasmid-partitioning protein ParB	−1.48	8.02E−10			
TRINITY_DN1855_c5_g1	Protein GrpE	−1.47	5.73E−04	Unfolded protein binding	Response to heat,	Cytoplasm
TRINITY_DN1289_c0_g1	Cell wall-associated hydrolase	−1.45	3.42E−03	Hydrolase activity		Membrane
TRINITY_DN3020_c0_g1	Glutamate synthase (NADPH) small chain	−1.44	1.11E−04	Protein binding, Fe–S cluster binding	Glutamate biosynthetic process	Cytosol
TRINITY_DN1794_c0_g1	Hypothetical protein AZ040_001793, partial	−1.44	1.74E−03			
TRINITY_DN234_c0_g1	Hypothetical protein	−1.41	5.00E−03			
TRINITY_DN1807_c0_g2	Hypothetical protein UTI89_C0218	−1.41	6.45E−03			
TRINITY_DN2089_c0_g1	Phospho-2-dehydro-3-deoxyheptonate aldolase	−1.40	1.69E−03	3-Deoxy-7-phosphoheptulonate synthase activity	Cellular amino acid process	Cytosol
TRINITY_DN1112_c0_g1	Hypothetical protein CDA59_06275	−1.38	4.15E−03			
TRINITY_DN1784_c0_g5	Hypothetical protein B1H11_05530, partial	−1.36	1.29E−02			
TRINITY_DN3649_c0_g1	Quinolinate synthase NadA	−1.35	2.75E−03			
TRINITY_DN1794_c1_g2	Hypothetical protein NTHI1209_00002	−1.33	5.72E−03			
TRINITY_DN1414_c0_g1	Copper-exporting P-type ATPase A	−1.32	4.73E−04	ATP binding, cation-transporting ATPase activity	Cation transport	Membrane
TRINITY_DN1807_c0_g4	Putative oRF58e	−1.31	1.30E−03			
TRINITY_DN1356_c0_g1	Conserved hypothetical protein	−1.30	7.82E−03			
TRINITY_DN3284_c0_g1	ATP-binding protein	−1.28	8.63E−02			
TRINITY_DN1376_c0_g1	Chaperone protein DnaJ	−1.28	2.52E−03	Protein binding, ATPase activity	Protein folding, DNA replication	Cytoplasm
TRINITY_DN1547_c0_g1	Hypothetical protein AC564_3197	−1.26	3.61E−03			
TRINITY_DN572_c0_g1	Copper-sensing transcriptional repressor CsoR	−1.25	3.81E−03	Metal ion binding	Response to copper, transcription regulation	Cytoplasm
TRINITY_DN1822_c0_g1	IS5 family transposase ISDra5	−1.23	3.93E−03			
TRINITY_DN4202_c0_g1	Pyridoxal 5′-phosphate synthase subunit PdxS	−1.20	4.15E−03	Catalytic activity	Vitamin B6 metabolic process	
TRINITY_DN3728_c0_g1	Anthranilate synthase component 1	−1.19	5.17E−03	Anthranilate synthase activity‘	Tryptophan biosynthetic process	Anthranilate synthase complex
TRINITY_DN1794_c1_g3	Hypothetical protein OXB_0978	−1.18	4.77E−03			
TRINITY_DN1260_c0_g2	IS4/IS5 family transposase	−1.18	9.13E−05			
TRINITY_DN136_c0_g1	Vitamin B12-dependent ribonucleoside-diphosphate reductase	−1.15	2.19E−04	Nucleotide, ATP binding	DNA replication	Membrane
TRINITY_DN165_c0_g1	Transposase for insertion sequence-like element IS431mec	−1.15	1.49E−04			
TRINITY_DN1621_c0_g1	Hypothetical protein	−1.13	7.02E−02			
TRINITY_DN1711_c6_g1	Hybrid sensor histidine kinase/response regulator	−1.12	3.34E−02	Protein binding, kinase activity	Phosphorelay, signal transduction	Cytoplasm, membrane
TRINITY_DN1354_c0_g1	Probable cytosol aminopeptidase	−1.11	3.23E−02			
TRINITY_DN54_c0_g1	Uncharacterized ABC transporter ATP-binding protein all4389	−1.08	3.42E−03			
TRINITY_DN1784_c1_g1	Hypothetical protein	−1.07	2.47E−02			
TRINITY_DN2112_c0_g1	Hypothetical protein	−1.07	5.93E−03			
TRINITY_DN1825_c0_g3	Cytochrome c oxidase subunit II	−1.07	5.40E−03	Cytochrome c oxidase activity	ATP synthesis, H transport	Membrane
TRINITY_DN3539_c0_g1	Glycerophosphodiester phosphodiesterase	−1.05	3.85E−03	Metal ion binding	Glycerol metabolic process	
TRINITY_DN1123_c0_g1	Type 4 prepilin-like proteins leader peptide-processing enzyme	−1.04	1.31E−03	Peptidase activity	Proteolysis	Membrane
TRINITY_DN1784_c2_g1	Hypothetical protein DR_0254	−1.03	9.14E−02			
TRINITY_DN3686_c0_g1	Pyruvate dehydrogenase E1 component subunit beta	1.01	3.42E−03	Catalytic activity	Metabolic process	
TRINITY_DN4098_c0_g1	Uncharacterized protein (DR_1649)	1.01	2.36E−03			
TRINITY_DN1463_c0_g1	Peptidase S41	1.03	4.67E−03	Serine-type peptidase activity	Proteolysis	
TRINITY_DN2060_c0_g1	Hexagonally packed intermediate-layer surface protein	1.07	2.37E−02		Cell wall organization	Cell wall
TRINITY_DN2278_c0_g1	Uncharacterized protein (DR_1425)	1.08	7.81E−02			
TRINITY_DN1557_c0_g1	ABC-type transport system, ATPase component/photorepair protein PhrA	1.09	1.49E−04	ATP binding, ATPase activity		
TRINITY_DN1640_c0_g2	Biopolymer transport protein, putative (DR_0456)	1.11	6.15E−02	Protein transporter activity	Transport	Membrane
TRINITY_DN1826_c1_g1	Peptide ABC transporter periplasmic peptide-binding protein	1.11	7.23E−03		Transmembrane transport	ABC transporter complex
TRINITY_DN1753_c0_g1	Uncharacterized protein (DR_1708)	1.13	1.26E−05			
TRINITY_DN1669_c0_g1	Signal peptidase I	1.16	1.86E−04	Peptidase activity	Signal peptide processing	Membrane
TRINITY_DN1167_c0_g1		1.19	4.71E−02			
TRINITY_DN1004_c0_g1	ABC transporter substrate-binding protein	1.33	2.37E−02		Transmembrane transport	ABC transporter complex
TRINITY_DN736_c0_g1	Uncharacterized protein (DR_1461)	1.36	2.86E−02			
TRINITY_DN4163_c0_g1	Branched-chain amino acid ABC transporter, permease protein	1.41	8.63E−02	Transporter activity	Transport	Membrane
TRINITY_DN2668_c0_g1	Probable ABC transporter-binding protein DR_1571	1.45	7.92E−02	Peptide transporter activity	Peptide transport	ABC transporter complex
TRINITY_DN413_c0_g1	Methylmalonyl-CoA mutase, alpha subunit, chain A	1.54	2.85E−02	Catalytic process	Metabolic activity	
TRINITY_DN3161_c0_g1	Autotransporter translocation and assembly factor TamB	1.54	7.44E−02			Integral component of the membrane
TRINITY_DN3046_c0_g1	Uncharacterized protein (DR_2599)	1.62	2.06E−02			
TRINITY_DN4297_c0_g1	Uncharacterized protein (DR_1465)	1.68	8.33E−04			
TRINITY_DN25_c0_g1	Cationic outer membrane protein OmpH, putative (DR_0989)	1.75	3.34E−02	Unfolded protein binding		
TRINITY_DN1832_c3_g2	SLH family protein	1.77	1.57E−06			Integral component of the membrane
TRINITY_DN653_c0_g1	Uncharacterized protein (CULT_160015)	1.80	3.34E−02			
TRINITY_DN1906_c0_g1	50S ribosomal protein L5	1.89	9.42E−04	tRNA binding	Translation	Ribosome
TRINITY_DN3573_c0_g1	S-layer protein SlpA	2.33	4.12E−12			Cell wall

### Gene Ontology Term Enrichment Analysis

GO term enrichment was performed only on libraries from cells harvested at 34 h, as 24-h samples only showed a small number of unigenes regulated, which is insufficient for this analysis (Figure 2A). The 34-h cells, corresponding approximately to late exponential phase, exhibited a clear distinction between cellular components responding to below background conditions. Various unigenes related to cytosol (GO:0005829), cytoplasm (GO:0005737), and membrane (GO:0016020) components were downregulated, while ABC transporter complex (GO:1990351), cell wall (GO:0005618), and ribosome (GO:0005840) genes were exclusively upregulated. Similarly, biological processes such as transport (GO:0006810) and transmembrane transport (GO:0055085) were upregulated, in contrast to protein transport (GO:0015031) and folding (GO:0006457), proteolysis (GO:0006508), regulation of transcription (GO:0006355), DNA replication (GO:0006260), and cation transport (GO:0006812) that were downregulated. We also observed the strong downregulation of metabolic functions such as ATP (GO:0005524), metal ion (GO:0046872), and protein (GO:0005515) binding.

## Discussion

These results, demonstrating the high degree of dissimilarity between these two “strains” of *D. radiodurans*, support the genetic analyses of Brooks et al. (1980) who documented only 33% DNA homology between the R1 and Sark strains. Because the R1 and Sark strains are likely to have less than 94% average nucleotide identities (a 94% has been proposed as defining a bacterial species, according to Konstantinidis and Tiedje, 2004), these two bacteria could be considered two separate species. This is a subject that needs formal taxonomic resolution, especially considering that these are two of the three representatives of *D. radiodurans* maintained at the American Type Culture Collection.

### Partial Loss of Proteome Integrity

The essential role of the *dnaJ*, *dnaK*, *clpB*, and *grpE* gene products in folding nascent or misfolded proteins, as well as processing protein aggregates is directly related not only to the maintenance of basic cellular processes (Ulrich, 1996; Calloni et al., 2012; Rosenzweig et al., 2013) but also to the capacity of the cells to respond to changes in their immediate environment (Feder and Hofmann, 1999; Guisbert et al., 2006). In our experiments, shielding cells from background radiation for 34 h resulted in a lower transcription rate of the unigenes (TRINITY_DN1376_c0_g1, TRINITY_DN1855_c4_g1, TRINITY_DN1782_c0_g1, and TRINITY_DN1855_c5_g1) corresponding to these proteins (Figure 2B), suggesting the potential intracellular accumulation of unfolded and/or aggregated proteins. These changes could have led to a slower growth rate and the subsequent difference in biomass accumulated during mid-exponential phase (Castillo et al., 2015). Similarly, the downregulation of DnaK in *Streptococcus mutants* caused a slower growth rate and an increased tendency to aggregate (Lemos et al., 2007), and the downregulation of DnaKJ was related to growth arrest on *Caulobacter crescentus* (Schramm et al., 2017), and a *Leptospira interrogans clpB* mutant exhibited a significant elongation of the lag phase along with an increased sensitivity to oxidative stress (Lourdault et al., 2011).

### Reduction in Nitrogen Assimilation

Bacteria assimilate nitrogen in the form of ammonium leading to the synthesis of the amino acids glutamine and glutamate, as well as precursor for pyrimidines and purines, among other molecules (van Heeswijk et al., 2013). The cellular components responsible for ammonium assimilation are controlled, primarily, by an ammonium membrane transporter (AmtB) and the enzymes involved in the assimilation pathways GS-GOGAT (glutamine synthetase-glutamate synthase) and GDH (glutamate dehydrogenase) (Zheng et al., 2004). Under NH_4_-limiting conditions, the GS-GOGAT dominates this process; therefore, the transcription of the genes for these enzymes has the potential to limit or stimulate growth (Hua et al., 2004). Specifically, GS catalyzes the conversion of glutamate into glutamine, while GOGAT reductively converts glutamine into two glutamate molecules using the tricarboxylic acid (TCA) intermediate 2-oxoglutarate as carbon structural component. This process constitutes an essential link between the carbon and nitrogen cycles (Commichau et al., 2006). Below-background radiation conditions exerted a repressive effect on the transcription of the unigenes corresponding to genes TRINITY_DN3594_c0_g1, TRINITY_DN1777_c0g2, TRINITY_DN1101_c0_g1/TRINITY_DN3020_c0_g1, and TRINITY_DN2597_c0_g1, coding for the ammonium transporter, glutamine synthetase, glutamine synthase, and the transcriptional regulator GlnK, respectively, potentially limiting the availability of amino acids and precursors for the synthesis of nucleic acids, resulting in diminished growth (Figure 2C).

### Copper Transport and Homeostasis

Copper plays several important roles in bacteria such as activity regulation of superoxide dismutase C and cytochrome C oxidase, allowing the cells to prevent the accumulation of toxic levels of superoxide (O_2_^–^) radicals and to transfer electron to O_2_ in the electron transfer chain, respectively (Osman and Cavet, 2008). In higher than physiological concentrations, however, copper induces toxicity *via* the accumulation of reactive oxygen species (ROS) through Fenton-like reactions (Grass and Rensing, 2001) and by disrupting Fe–S clusters, essential in electron transfer reactions (Chillappagari et al., 2010). For this purpose, bacteria tightly regulate the intracellular concentration of copper using transmembrane transport proteins to efflux Cu/Cu^2+^ ions (Puig and Thiele, 2002). The absence of background levels of radiation during the growth of *D. radiodurans* induced the downregulation of four unigenes related to copper transport and homeostasis (TRINITY_DN1414_c0_g1; TRINITY_DN572_c0_g1, TRINITY_DN1311_c0_g1, and TRINITY_DN610_c0_g1), suggesting a potential partial impairment of cells to prevent the accumulation of toxic levels of intracellular copper, inducing an oxidative stress state.

### Increased Protein Transport and Cell Wall Assembly

Shielding of cells from background radiation caused the upregulation of two unigenes (TRINITY_DN1463_c0_g1 and TRINITY_DN1669_c0_g1) related to protein transport and secretion by the general secretory pathway (Waite et al., 2012; Carroll et al., 2014). The proteins coded by these unigenes have different functions such as the acquisition of nutrients and intercellular communication (Gerlach and Hensel, 2007), suggesting that our treatment triggered these two functions most likely as a resource to maintain optimal growth. Similarly, TRINITY_DN3161_c0_g1 and TRINITY_DN3573_c0_g1, coding for proteins involved in the assembly of autotransporters and cell envelop synthesis, resistance to shear, and osmotic stress, as well as prevention of oxidative stress (Sleytr and Beveridge, 1999; Selkrig et al., 2012), respectively, were overexpressed. The joint effect of these processes, however, was insufficient to overcome the deleterious effects of the multiple downregulated systems previously discussed.

### Gene Expression Regulation in Two Taxonomically Dissimilar Bacteria: *Deinococcus radiodurans* vs *Shewanella oneidensis*

**FIGURE 3:**
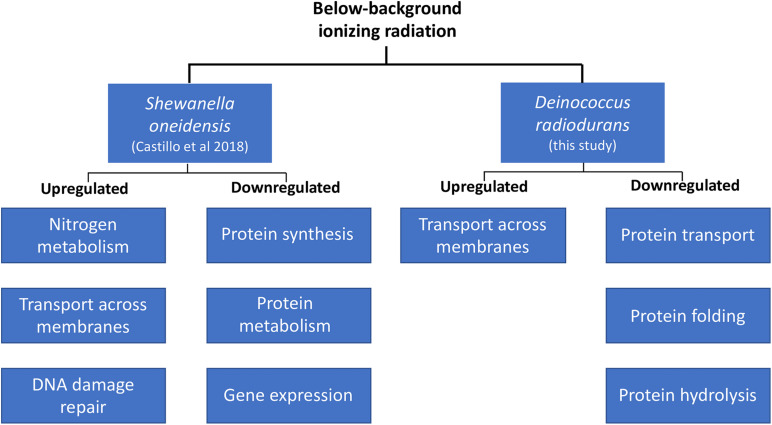
Transcriptional regulation in *D. radiodurans* and *Shewanella oneidensis* in response to below-background ionizing radiation levels based on biological processes.

A comparison of the biological processes enriched in *D. radiodurans* (this study) and *S. oneidensis* (Castillo et al., 2018) shows some similarities in their response to our treatment, despite their physiological differences (Figure 3). For instance, both species upregulated the expression of genes related to the transport of substrates (e.g., macromolecules, ions, complexes, and organelles) across the cell membrane and decreased the transcription of genes involved in various aspects of protein synthesis, transport, and activity. Our data shows that both species perceive radiation as an environmental cue. For instance, the extension of the lag phase and the consequent lower biomass accumulation in *D. radiodurans* differs from the lack of response in *S. oneidensis*’ growth dynamics (Castillo and Smith, 2017). In contrast, *S. oneidensis* exhibited a strong, coordinated response in terms of gene regulation (Castillo et al., 2018), while *D. radiodurans* regulated a significantly lower number of genes at a comparable growth stage. In broad terms, both species responded to our treatment as they do to different types of stress. *S. oneidensis* responded strongly to it, while *D. radiodurans* did not, resulting in its modulation of growth. An argument could be made on both models’ widely documented sensitivity to ionizing radiation. *D. radiodurans*, among all organisms on Earth, possess the highest resistance to ionizing radiation (D_10_ 10–12 kGy), while *S. oneidensis* is considered as one of the most sensitive organisms (D_10_ 0.07 kGy) within the prokaryotic domain (Ghosal et al., 2005). Could *D. radiodurans*’ outstanding capacity to withstand the deleterious effects of ionizing radiation cause a strong dependence on some of the ROS products formed by the lysis of water? Could *S. oneidensis*’ sensitivity to this same stress result in cells prone to a more efficient genome expression regulation in order to retain homeostatic control? It is known that in the cellular world, a common survival strategy to face unfavorable conditions is the prioritization of similar metabolic activities, such as the SOS response elicited by DNA damage. If ionizing radiation is indeed a catalyzer for the production of ROS essential for transcriptional control, it would be expected that cells shielded from it would undergo a transient “shortage” of specific gene expression initiators. This study adds to the body of knowledge about the molecular effects of below background levels of IR and supports the hypothesis of a biological role of reactive oxygen species on the homeostatic control of cells.

## Data Availability Statement

The datasets presented in this study can be found in online repositories. The names of the repository/repositories and accession number(s) can be found below: https://www.ncbi.nlm.nih.gov/, PRJNA389981.

## Author Contributions

GS conceived and designed the study, reviewed and edited the manuscript. GS and HC ran the experiments and collected the field data. HC extracted the nucleic acids and constructed the RNA libraries. XL and HC performed the bioinformatic analysis and wrote the manuscript. All authors read and approved the final manuscript.

## Conflict of Interest

The authors declare that the research was conducted in the absence of any commercial or financial relationships that could be construed as a potential conflict of interest.

## Footnotes

1 http://www.bioinformatics.babraham.ac.uk/projects/fastqc/

2 https://www.ncbi.nlm.nih.gov/tools/vecscreen/about/

3 http://weizhongli-lab.org/cd-hit/

4 https://github.com/Trinotate/Trinotate.github.io/wiki
