# Supplementary material for: Deinococcus radiodurans UWO298 Dependence on Background Radiation for Optimal Growth
Source: Front Genet. 2021 May 6;12:644292. doi: 10.3389/fgene.2021.644292 (PMC8136434; doi:10.3389/fgene.2021.644292)
Supplement: Supplementary file 1 [file Data_Sheet_1.docx]

**Supplementary table 1 (ST1). Primers used for the qPCR validation of the present study transcriptome analysis.**

**Supplementary table 1 (ST2). RNASeq libraries sequencing data and quality control processing.**

**Supplementary table 3 (ST3). RNASeq libraries’ assembly statistics.**
